# Integrated Dynamics of Kinetic and Kinematic Control in Hand Grip Function: Postural and Visual Effects

**DOI:** 10.1055/s-0045-1810042

**Published:** 2025-08-18

**Authors:** Bernardo Figueira Althoff, João Carlos Nakamoto, Mateus Saito, Luiz Sorrenti, Ricardo Boso Escudero, Erick Yoshio Wataya

**Affiliations:** 1 Instituto Vita, São Paulo, SP, Brazil

**Keywords:** feedback, movement, wrist, movimento, punho, retroalimentação

## Abstract

**Objective:**

To interpret, with objective data, the kinetic and kinematic control of gripper function associated with visual and stereognostic control.

**Methods:**

In total, 34 young participants, without previous hand diseases or traumas, underwent pinch grip tasks with the wrist in a neutral position and at 45° of flexion. The tasks were repeated three times.

**Results:**

The movement difference in the neutral and 45° of flexion postures presented a significant correlation with the pulp-to-pulp distance variables (r values from 0.38 to 0.41; *p* < 0.05). The strength difference in the neutral and 45° of flexion postures, with or without visual feedback, also showed a significant correlation (r values from 0.45 to 0.47; *p* < 0.01). The movement and strength differences presented a significant correlation in the neutral posture without visual feedback (r = 0.77; *p* < 0.001) and in the flexed posture with visual feedback (r = 0.48; *p* = 0.004).

**Conclusion:**

Visual feedback and wrist posture influence strength and movement control in gripping function in healthy adults. These findings reinforce the interdependence of control mechanisms in hand function. Postural adjustments and proprioception enhancement can optimize functional recovery, with implications for the development of specific tests and their application in actual clinical settings.

## Resumo

**Objetivo:**

Interpretar, com dados objetivos, o controle cinético e cinemático na função de pinça associado ao controle visual e estereognóstico.

**Métodos:**

Ao todo, 34 participantes jovens, sem doenças ou traumas prévios nas mãos, foram submetidos à aplicação de tarefas com pinça, com o punho em posição neutra e fletida a 45°. As tarefas foram repetidas três vezes.

**Resultados:**

A diferença do movimento nas posturas neutra e flexionada a 45° foi significativamente correlacionada às variáveis de distância polpa-a-polpa (valores de r entre 0,38–0,41; *p* < 0,05). A diferença da força nas posturas neutra e flexionada a 45°, com ou sem *feedback* visual, também apresentou correlação significativa (valores de r entre 0,45–0,47; *p* < 0,01). A diferença do movimento foi significativamente correlacionada com a diferença da força na postura neutra sem *feedback* visual (r = 0,77; *p* < 0,001) e na postura flexionada com *feedback* visual (r = 0,48; *p* = 0,004).

**Conclusão:**

O controle de força e de movimento na função da pinça em adultos saudáveis está relacionado ao *feedback* visual e à postura do punho. Esses achados reforçam a interdependência dos mecanismos de controle na função manual. Ajustes posturais e o aprimoramento da propriocepção podem otimizar a recuperação funcional, com implicações para o desenvolvimento de testes específicos e sua aplicação em ambientes clínicos reais.

## Introduction

The human hand is a complex organ composed of bones, muscles, tendons, fascia, ligaments, nerves, and blood vessels under a uniquely loose dorsal skin and glabrous palmar skin. The extreme adaptability of the hands to objects and tasks represents years of evolution. The ability to grasp and manipulate objects is fundamental to human progress, whose fate was shaped by tool manipulation. 1 2 Physiologically, the hand is the “performing extremity” because it enable the adoption of numerous positions to perform several functions. From a kinetic and kinematic perspective, the functional complexity of the human hand provides a range of potential positions, movements, and actions. The gripping function reaches levels not seen in other animals due to the peculiar position of the thumb, which can oppose all other fingers. 3 The present study aimed to interpret, using objective data, gripping control during force application and the distance between the thumb and index finger pulps in the presence or absence of visual and stereognostic feedback under different wrist positions. Such information can provide insights for clinical interventions, rehabilitation, and ergonomic design.

## Materials and Methods

**Participants:** We included young participants, aged 20 to 40 years, without comorbidities or known hand conditions. The exclusion criteria were subjects with any previous hand surgery, compressive syndromes affecting the peripheral nerves of the upper limb, systemic inflammatory arthritis, and osteoarthritis of the hand and wrist joints, and those who refused to sign the informed consent form. The ethics committee of our institution approved the study under number CAAE 66899023.2.0000.5474.

**Fig. 1:**
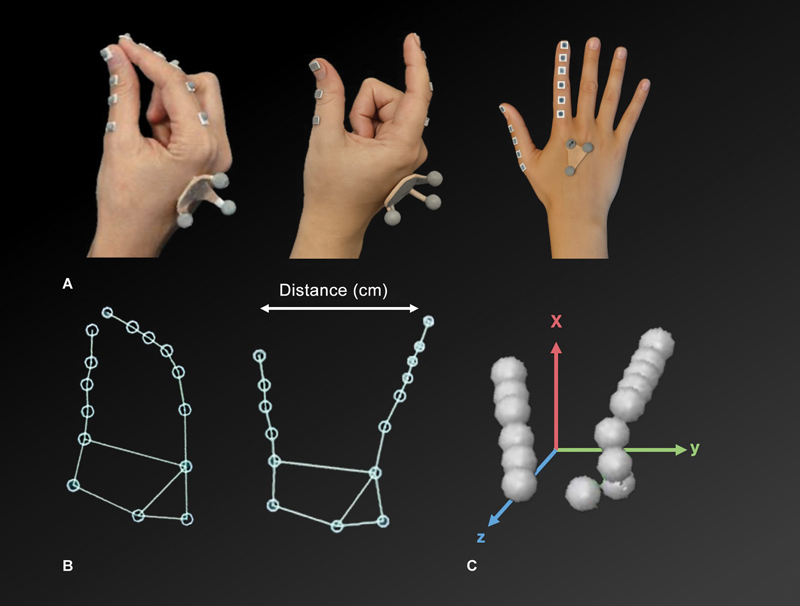
Movement data collection: Capture system using reflective passive markers recorded by infrared cameras positioned around the test area. ( **A** ) Marker positioning on the back of the hand, thumb, and index finger. ( **B** ) Two-dimensional reconstruction of the hand with marking of the tip-to-tip distance (in centimeters). ( **C** ) Three-dimensional reconstruction with indication of the X (red), Y (green), and Z (blue) spatial axes.

**Movement and pulp-to-pulp distance data collection:** We determined the pinch of pulp of the index finger with the pulp of the thumb. We used a system with passive markers captured by infrared cameras positioned around the test area to collect data on the movements of the thumb and index finger during the long pinch movement task on the X (lateral), Y (vertical), and Z (depth) axes and measure the three-dimensional kinetics. Reflective markers placed at strategic points (two on each phalanx of the thumb and index finger) enabled movement analysis in three dimensions ( Fig. 1 ).

**Fig. 2:**
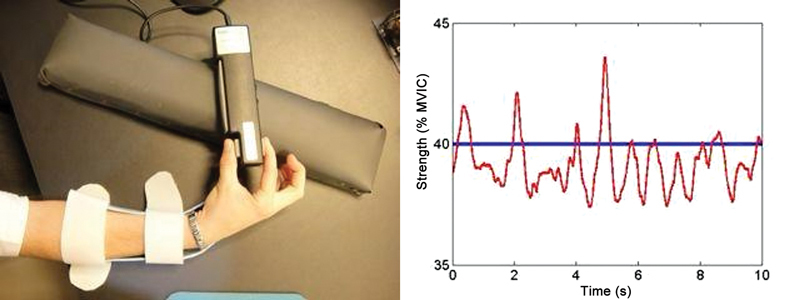
Force data collection: Dynamometry system for grip with digital transducer (MedEOR MedTech) and measurement software. **Abbreviation:** MVIC, maximum voluntary isometric contraction.

**Pulp-to-pulp strength data collection:** A dynamometer gripping system with a digital transducer (MedEOR MedTech) and software performed real-time translation of the force level and target into visual graphs to measure the gripping force ( Fig. 2 ).

The distance variation between the tips of the thumb and index finger in pinch closures (resulting pulp-to-pulp distance and pulp-to-pulp distance in each direction with index finger maximum extension and maximum thumb extension and abduction) and strength variability in the isometric pinch task were described by the standard deviation of each time series and used as performance indicators during the requested tasks.

## Tests

We asked 34 young participants without previous hand diseases or traumas to perform the following two tasks:

- Precision pinch movement: Performed with the thumb and index finger, the participants made the pinch movement at a 2-second rhythm for each 25-second cycle in 2 positions: with the wrist at a neutral position, with and without visual feedback, and with the wrist at 45° flexion, with and without visual feedback.
- Precision pinch isometric strength control: The participants used their thumb and index finger to apply 40% of their maximum voluntary isometric strength for 25 seconds (3–5 N) in 2 positions: with the wrist at a neutral position, with and without visual feedback, and with the wrist at 45° flexion, with and without visual feedback.

We removed the visual feedback after the participant reached and maintained the target level of 40% of maximum voluntary strength. Each participant repeated the test three times. We described the variation in the distance between the fingertips (resultant tip-to-tip distance and tip-to-tip distance in each direction) and the strength variation in the isometric pinch task as standard deviation values for each time series and used it as performance indicators during the two tasks. The Pearson correlation coefficient (r) determined the correlation between these performances with a statistical significance level ( *p* ) of 0.05.

## Results

For the pinch movement variability in three axes (X, Y, Z), only the X axis presented a significant correlation with the total tip-to-tip pinch movement. In the neutral position with the eyes open, we identified an average pinch force of 4.95 N and a tip-to-tip distance of 12.2 cm. In the same position with closed eyes, the force increased to 5.42 N, while the distance increased to 13.4 cm. In the flexed position with eyes open, the average force decreased to 4.68 N, and the distance was of 12.7 cm, indicating potential mechanical limitations imposed by wrist flexion. In the flexed position with eyes closed, the force was of 5.05 N, and the distance reached 13.9 cm ( Tables 1 2 ).

**Table 1:** Mean tip-to-tip distance under different experimental conditions

Gripping distance (cm)		
	Eyes open	Eyes closed
Neutral position	12.2	13.4
Flexed position	12.7	13.9

**Table 2:** Mean grip strength under different experimental conditions

Grip strength (N)		
	Eyes open	Eyes closed
Neutral position	4.95	5.42
Flexed position	4.68	5.05

The study revealed variable correlation coefficients, highlighting a significant interaction between movement and strength control. In the neutral wrist position without visual feedback, the correlation between movement and force variability was significantly strong (r = 0.77; *p* < 0.001). In contrast, in the flexed wrist position with visual feedback, the correlation was moderate (r = 0.48; *p* = 0.004). The absence of visual feedback significantly impacted the correlation between force variability and pulp-to-pulp distance, with r = 0.73 and *p* = 0.025 in the neutral position. Additionally, we observed a significant correlation (r = 0.59; *p* = 0.015) between force without visual feedback in the neutral position and with visual feedback in the flexed position. The analyses also indicated significant correlations in the movement and force differences between the neutral and flexed positions, with coefficients ranging from 0.38 to 0.41 ( *p* < 0.05) for movement and from 0.45 to 0.47 ( *p* < 0.01) for force. These results highlight the interactions of experimental conditions and the pinch function, demonstrating variations in the correlation based on wrist posture and the presence of visual stimuli ( Tables 3 4 ).

**Table 3:** Correlations between force and movement in the neutral and flexed positions

Correlation (r)					
	TTdX	FN-OE	FN-CE	FF-OE	FF-CE
**FN-OE**	0.56				
**FN-CE**	0.73	0.83			
**FF-OE**	0.6	0.84	0.79		
**FF-CE**	0.65	0.59	0.6	0.51	

**Abbreviations:** CE, closed eyes; FF, force in the flexed position; FN, force in the neutral position; OE, open eyes; TTdX, tip-to-tip distance on the X axis.

**Table 4:** Statistical significance in the correlations between force and movement in the neutral and flexed positions

Statistical significance ( *p* -value)					
	TTdX	FN-OE	FN-CE	FF-OE	FF-CE
**FN-OE**	0.025	1			
**FN-CE**	0.001	0	1		
**FF-OE**	0.013	0	0	1	
**FF-CE**	0.006	0.015	0.014	0.044	1

**Abbreviations:** CE, closed eyes; FF, force in the flexed position; FN, force in the neutral position; OE, open eyes; TTdX, tip-to-tip distance on the X axis.

## Discussion

The terminal tip-to-tip opposition pinch is the most precise grip, requiring adequate opposition of the thumb and index finger and integrity of the digital pulps, joints, tendons, and muscles involved, especially the flexor digitorum profundus muscle of the second finger and the flexor pollicis longus. 4 In addition to being an executor, the hand is a highly-sensitive somatosensory organ, essential for spatial, tactile, and stereognostic perception, enabling object recognition without the need for direct vision. 5 In the current study, we observed that the wrist position and the presence or absence of visual feedback significantly influence grip precision and strength, demonstrating the adaptability of hand motor control mechanisms under different sensory and mechanical conditions:

**Variable correlation:** We observed consistency in the correlations between pinch distance and force across different conditions, indicating that subjects sustain a control pattern influenced by visual feedback and wrist position. This pattern suggests that pinch manipulation is adaptive, adjusting to maintain efficacy in the face of sensory and mechanical changes imposed by different experimental conditions.

**Visual feedback effect:** The absence of visual feedback increased the applied force and the pinch distance. The correlation coefficients for force (r = 0.51 and 0.83; *p* < 0.05) and distance (r = 0.56 and 0.73; *p* < 0.05) illustrate that, in the absence of visual feedback, participants tend to apply more force and reach a greater pulp-to-pulp distance. This increase can be an attempt by the subjects to compensate for the lack of visual information, ensuring precision in the pinch manipulation.

**Influence of the flexed position:** Wrist flexion significantly reduced pinch force, decreasing from 4.95 N to 4.68 N with eyes open (r = 0.60; p = 0.013). With eyes closed, the force slightly increased to 5.05 N (r = 0.51; p = 0.044), suggesting an adaptive response to the lack of visual input. These findings indicate that wrist flexion imposes mechanical constraints that may be partially compensated by sensory mechanisms activated in the absence of visual stimuli.

**Correlation between movement variability and strength control:** There was a notable correlation between movement and force variabilities, with a significant association, especially in the neutral position without visual feedback (r = 0.77; *p* < 0.001) and in the flexed position with visual feedback (r = 0.48; *p* = 0.004). This finding reinforces the concept of movement and force interconnection and that visibility and mechanical wrist position modulate this relationship.

Gripping relies on the application of effective forces by the hand to perform tasks, 6 requiring precise force and movement control. As such, understanding the relationship between pinch kinetics and kinematics is essential to interpret functional and pathological hand abnormalities. Li et al. 7 investigated the effects of hand dominance on finger force variability during precision pinch, observing that the absence of visual feedback increases variability, and that the dominant hand presents better precision and coordination. Conditions also influence this function, as demonstrated by Nataraj et al., 8 who reported that carpal tunnel syndrome reduces the range of motion and increases pinch variability, affecting trajectories, joint angles, and digital contact, impairing dexterity and precision.

It is worth noting that the current study has some limitations. First, the sample size was relatively small, which may limit the generalizability of the results to other populations. In addition, the study was conducted under controlled laboratory conditions, which may not reflect natural and clinical scenarios.

These results enable an analytic inference of a correlation between grip kinetic and kinematic controls, which did not occur independently in the population studied. The relationship between movement and force in pinch grip suggests the interconnection of these two processes and the interdependence of the control mechanisms in hand function.

## Conclusion

The present study demonstrated that pinch grip function control regarding force and distance between the fingers undergoes a significant influence by wrist position and the presence of visual feedback. The neutral position favored better precision and less variability, while flexion increased variability and reduced control, especially without visual feedback. These findings confirm the interdependence of pinch kinetic and kinematic mechanisms, as proposed in the study objective. Thus, interventions considering wrist position and sensory training can improve hand function, with relevant applications in the clinical practice, rehabilitation, and ergonomic settings. Future research can develop specialized tests to evaluate precision pinch in selected diseases, validating the results in large populations and real-life scenarios to improve clinical interventions.
